# Assessment of patient expectations toward health professionals and quality of life among Płock (Poland) residents aged 55 and over: a cohort study

**DOI:** 10.3389/fpubh.2024.1434693

**Published:** 2024-11-28

**Authors:** Mariola Głowacka, Monika Gasik, Waldemar Kujawa, Mateusz Cybulski, Agnieszka Pluta

**Affiliations:** ^1^Collegium Medicum, The Mazovian University in Płock, Płock, Poland; ^2^Provincial Polyclinical Hospital in Płock, Płock, Poland; ^3^Faculty of Medicine, Collegium Mediucm, Mazovian University in Płock, Płock, Poland; ^4^Department of Integrated Medical Care, Faculty of Health Sciences, Medical University of Bialystok, Bialystok, Poland; ^5^Department of Preventive Nursing, Faculty of Health Sciences, Collegium Medicum in Bydgoszcz, Nicolaus Copernicus University in Toruń, Torun, Poland

**Keywords:** older adults people, health behaviours, older adults, patient request, quality of life

## Abstract

**Background:**

The right attitude of health professionals (mainly doctors) towards patients—particularly older ones—determines patients’ level of illness acceptance and quality of life. The aim of the study was to assess patient expectations of health professionals and quality of life among residents of Płock (Poland) aged 55 and over in relation to sociodemographic variables and to examine correlations between these patients’ expectations and their quality of life.

**Methods:**

The study included 2040 adults aged 55 and over, with 68.9% being women. A diagnostic survey was used, incorporating sociodemographic questions and two standardized scales: the Patient Request Form (PRF) and the 36-Item Short Form Health Survey (SF-36). This cohort study utilized a multi-stage sampling method, with data collected through a diagnostic survey. Data analysis incorporated ANOVA, post-hoc Fisher’s LSD tests, and Pearson’s correlation to assess relationships between variables.

**Results:**

Statistically significant differences were observed between male and female respondents in their expectations of health professionals. Women reported higher scores than men across all three PRF scales, including emotional support (4.85 vs. 4.35, *p* < 0.05), test- and treatment-related information (7.41 vs. 6.81, p < 0.05), and explanation of the illness (6.89 vs. 6.47). A low but statistically significant correlation was found between age and expectations for emotional support (r = 0.162, *p* < 0.001), as well as test- and treatment-related information (r = 0.122, *p* < 0.001). Respondents with secondary/post-secondary education reported the highest expectations for explanation of the illness (mean = 7.06) and test- and treatment-related information (mean = 7.64). Meanwhile, respondents with lower education levels, especially those with primary or vocational education, reported the highest expectations for emotional support (mean = 4.93). The average QoL score measured by the SF-36 was 63.45 (SD = 17.68), indicating moderate-to-high overall QoL. The mental component summary score (mean = 65.07, SD = 19.69) was slightly higher than the physical component summary score (mean = 62.70, SD = 18.06). Age was negatively correlated with QoL scores, particularly in the physical component (r = −0.407, *p* < 0.001). Education level positively influenced QoL, with respondents holding tertiary education reporting the highest QoL scores.

**Conclusion:**

What the older adults included in our study expect most from health professionals is full and ac-curate medical information, particularly information about their health. Overall, the respondents reported moderate, bordering on high, quality of life. The quality of life of the respondents and their expectations of health professionals were influenced by sociodemographic characteristics, and in particular sex, age and education level.

## 1 Introduction

Nowadays, older people are a growing population with special needs related to significant advancements in science and technology, including in particular developments in medical diagnostic and treatment methods, and growing health awareness among people in this age group (1). The increasing proportion of older people in society poses a challenge for public health (2). As in many other countries around the world, the population of Poland is ageing rapidly. The latest demographic figures show that more than 6.7 million (17.5%) people in Poland are aged 65 or over (3). According to projections, by 2060 Poland will have one of the oldest populations in Europe (4). Płock, located approximately 115 km from Warsaw, had a population of 113,660 as of December 31, 2022. Among its older adults residents, females represent over 67% of the population aged 65 and older for men, and 60 and older for women (5, 6). This demographic imbalance highlights significant challenges for healthcare services, particularly in addressing the differing health expectations and needs of the predominantly female older adults population. These demographic shifts pose a considerable challenge to public health infrastructure, particularly in smaller cities like Płock, where the demand for healthcare services for older adults is rising (7).

Quality of life is an important factor in determining health status; in the case of older individuals, a number of physical aspects of quality of life, such as the energy to be physically active, absence of pain and ability to perform daily activities, are particularly important. The extent to which individual domains of quality of life are affected varies and depends on the environment, preferred lifestyle and degree of decline in capacities (8, 9). The concept of quality of life can be analysed from different perspectives, viewing it as multidimensional (9) in such a way that it can only be measured and described in individual terms of both objective circumstances and subjective assessments. The latter depend on the assessments and interpretations made by individuals about particular situations and conditions of the environment in which they function and on the individuals’ attitude towards adverse situations and their satisfaction with themselves and their lives (9, 10). A distinction has been made between general quality of life and health-related quality of life (HRQoL). The term ‘general quality of life’ encompasses an individual’s wellbeing and happiness and does not refer to health problems or other disorders, whereas HRQoL is part of a multidimensional approach where special focus is given to the functional consequences of the illness and its treatment for physical, psychological and social functioning (11). Understanding the associations between biological ageing, factors specific to individuals and the dimensions of quality of life is important for planning services and interventions to enhance quality of life in older individuals (12).

Given their health needs, older adults require regular appointments with health care professionals. The right attitude of health professionals (mainly doctors) towards patients—particularly older ones—determines, among other things, the patients’ level of illness acceptance and quality of life. Therefore, interactions between a patient and a health professional should include, in addition to the direct provision of health care services, provision of information about the patient’s health status, course of their illness, its treatment and types of possible side effects and, most importantly, should result in the establishment and maintenance of emotional contact through empathy and kindness in direct relations between the patient and the health professional. The quality of the interactions is important in the recovery process as it plays a major role in helping the patient develop the right attitude to their illness and treatment and has an impact on the patient’s mental wellbeing and motivation to manage their illness. Active contact between the patient and their doctor helps the patient feel more secure and place more trust in their physician and thus increases the patient’s motivation to adhere to treatment (13, 14). Studies on patients’ expectations of health professionals are an important but underrated niche within research on the quality of life of older adults. One issue that has not been examined and described in detail in the literature so far is the experience of older individuals with healthcare professionals, and in particular the attitude of health professionals towards older patients. To our knowledge, this is the first study on the subject in Poland to include such a large cohort of respondents and examine relationships between older patients’ expectations of health professionals and their quality of life.

While the general concept of quality of life among older adults has been explored, little attention has been given to the specific expectations older patients have towards healthcare providers, particularly regarding communication, empathy, and provision of medical information (5, 8, 9). The aim of the study was to assess patient expectations of health professionals and quality of life among residents of Płock (Poland) aged 55 and over in relation to sociodemographic variables, such as sex, age and education, and to examine correlations between patients’ expectations and their quality of life. The specific expectations assessed in this study include communication, empathy, professionalism, and the provision of medical information, measured using the Patient Request Form (PRF), alongside health-related quality of life (HRQoL) indicators. The cohort study design was chosen as it allows for the comprehensive analysis of the relationships between patients’ expectations and quality of life over time, capturing both sociodemographic influences and potential changes in health perceptions.

## 2 Materials and methods

### 2.1 Participants and study design

The study employed a cross-sectional design, where data were collected at a single point in time without longitudinal follow-up of participants. The study was conducted between January and November 2022. Respondents were recruited in three stages. In the first stage, 2,253 individuals gave their consent to participate in the study. Participants were recruited from the members of the Płock University of the Third Age and patients of primary health care surgeries in Płock—a town of 113,660 inhabitants (as at 31 December 2022) in the Mazovia Province located approximately 115 km from Warsaw, capital of Poland (12). The minimum required sample size for our study was determined to be 383, taking into account the available population and a 95% confidence level. This calculation was based on demographic data from Poland up to the year 2020 (6, 7). In the second stage, the Mini Mental State Examination (MMSE) was performed on all participants. All individuals with an MMSE score of between 27 and 30 (*n* = 2,102; 93%), indicating the absence of cognitive impairment, were included in the next (proper) stage of the study. The inclusion criteria were as follows: age ≥ 55 years; individuals residing or domiciled in Płock; absence of cognitive impairment (as assessed by the MMSE).

All individuals who met all the inclusion criteria chose whether to complete the survey on paper, at the place where they were recruited or at home, or electronically. The electronic survey was prepared and hosted on the LimeSurvey platform (LimeSurvey GmbH, Hamburg, Germany, with responses securely transmitted using encrypted connections to protect participant privacy. Paper surveys were manually entered into the system by authorized personnel, ensuring consistency and confidentiality during data transfer. All data were stored on secure servers with restricted access, limited to designated research staff. To ensure participant confidentiality, the data were anonymized prior to analysis, and only aggregate, non-identifiable data were used in the final analysis. We then reviewed the questionnaires received for completeness. Incomplete questionnaires were excluded from the analysis. A total of 2,040 fully completed questionnaires (97%), 1,406 of which (68.9%) were completed by women, were included in the analysis. The study employed a cohort design, with data collected at a single point in time without longitudinal follow-up.

### 2.2 Measures

The study was conducted using a diagnostic survey by means of our own survey questionnaire including sociodemographic questions and two standardised psychometric scales: the Patient Request Form (PRF) and the 36-Item Short Form Health Survey (SF-36).

The PRF is a self-report instrument. Its Polish version consists of 18 statements about the reasons for wanting to visit a general practitioner. Respondents are asked to rate each statement for how much it applies to their visit to their doctor. The statements are aggregated into the following three factors: expectations for explanation of the illness, expectations for emotional support and expectations for test- and treatment-related information. Expectations for explanation of the illness are related to seeking assistance from specialist doctors, whereas expectations for test- and treatment-related information are related to seeking assistance from health services. Expectations for emotional support are related to attaching importance to psychological and psychiatric counselling and assistance. Respondents are asked to rate how much each statement applies to their visit by choosing one of three answers: ‘agree’, ‘uncertain’ and ‘disagree’. All statements must be rated. The questionnaire takes on average not more than 10 min to complete. ‘Agree’ is scored as 2, ‘uncertain’ is scored as 1 and ‘disagree’ is scored as 0. The questionnaire yields a separate score for each of its three scales. Each scale has a possible score range of 0 to 12. The higher the score, the higher the level of expectation for a given type of assistance (15, 16).

The 36-Item Short Form Health Survey (SF-36) is a self-report instrument designed to measure subjective health status. It consists of 11 questions and 36 items generating 8 dimensions: physical functioning (I), role limitations due to physical health problems (II), bodily pain (III), general health (IV), vitality (V), social functioning (VI), role limitations due to emotional problems (VII) and mental health (VIII). The dimensions can be aggregated into two summary measures—the physical component summary scale (dimensions: I, II, IV, VIII; maximum possible score: 103) and the mental component summary scale (dimensions: III, V, VI, VII; maximum possible score: 68). The instrument produces a total SF-36 score (maximum possible score: 171) which is calculated as the sum of all 8 dimension scores and is a measure of overall health status. The higher the score, the higher the quality of life. For the purpose of comparative analyses, scores on the SF-36 were transformed into a 0–100 scale. The internal consistency of the subscales of the SF-36 for the general population is 0.78–0.93 (17–20).

### 2.3 Procedure and ethical considerations

The study was carried out in accordance with recommendations and was reviewed and approved by the Bioethics Committee of the Mazovian Academy in Płock (statute no. KB/N/BN/P/1.2021). All participants gave their written informed consent in accordance with the Declaration of Helsinki.

### 2.4 Statistical analysis

Statistica 10.0 (StatSoft Polska Sp. z o.o., Kraków, Poland) and PQStat (PQStat Software, Poznań, Poland) were used to analyse the data collected. Descriptive statistics for continuous variables were reported as means (M), standard deviations (SD), medians (Me), minimum values (Min.), maximum values (Max.), lower quartiles (Q_25_) and upper quartiles (Q_75_). Categorical variables were reported as frequencies (N) and percentages (%). Differences in one variable between three or more groups were examined using one-way ANOVA. Fisher’s LSD post-hoc test was used to determine which groups differed. Relationships between quantitative variables were tested using Pearson’s correlation. Statistical significance was set at *p* < 0.05.

## 3 Results

### 3.1 Respondents’ characteristics

The study included 2,040 respondents, the majority of whom were women (68.9%). The mean age of respondents was 65.4 years, with men being slightly older than women (66.87 vs. 64.8 years, *p* < 0.001). The largest proportion of respondents were aged between 60 and 75 (52.6%), and the smallest proportion were over 90 (1.2%). Most respondents had secondary or basic vocational education (56.4%), while a small fraction had no or incomplete primary education (2.4%). The majority of respondents were retired (54.5%) or employed (34.7%), with the smallest groups reporting self-employment or other specific statuses (0.2% or less). The mean duration of employment was 35.0 years, significantly longer for men than women (37.8 vs. 33.8 years, *p* < 0.001). Table 1 provides detailed sociodemographic characteristics of the respondents.

**Table 1:** Respondents’ sociodemographic characteristics.

	Sociodemographic characteristic	*n*	%
Sex	Female	1,406	68.9
	Male	634	31.1
Age	60 or under	664	32.5
	61–65	319	15.6
	66–70	460	22.5
	71–75	294	14.4
	76–80	173	8.5
	81–85	84	4.1
	86–90	22	1.1
	Over 90	24	1.2
Education	No	38	1.9
	Incomplete primary	11	0.5
	Primary	126	6.2
	Basic vocational	567	27.8
	Secondary	584	28.6
	Post-secondary	228	11.2
	Tertiary vocational (B.Sc., B.A. or B.Eng. degree)	172	8.4
	Master’s degree	314	15.4
Social and professional status	Old-age pensioner	1,112	54.5
	Disability pensioner	90	4.4
	Unemployed	27	1.3
	Running a household	55	2.7
	Employed	707	34.7
	Self-employed	4	0.2
	Disability pensioner, employed	4	0.2
	Old-age pensioner, employed	36	1.8
	Old-age pensioner, disability pensioner	4	0.2
	Recipient of a rehabilitation benefit	1	0.05
Source of income	Manual occupation	393	19.3
	Non-manual occupation	375	18.4
	Job outside agriculture	8	0.4
	Agricultural job	95	4.7
	Full-time employment	228	11.2
	Part-time employment	28	1.4
	Old-age pension	831	40.7
	Disability pension	61	3.0
	Other	16	0.8
	Old-age pension and disability pension	5	0.2

### 3.2 Patients’ expectations of health professionals (PRF)

Respondents reported the highest scores for expectations for test- and treatment-related information (M = 7.22), and the lowest scores for expectations for emotional support (M = 4.69). Descriptive statistics for scores for the three types of expectations of health professionals (factors) measured by the PRF are shown in Table 2.

**Table 2:** Descriptive statistics for the PRF.

Factor	*N*	M	SD	Min.	Max.	Q25	Me	Q75
Expectations for explanation of the illness	2040	6.76	4.638	0.0	12.0	1.0	8.0	11.0
Expectations for emotional support	2040	4.69	4.263	0.0	12.0	0.0	4.0	8.0
Expectations for test- and treatment-related information	2040	7.22	4.809	0.0	12.0	2.0	9.0	12.0

M, arithmetic mean; SD, standard deviation; Min., minimum value; Max., maximum value; Q_25_, lower quartile; Q_75_, upper quartile; Me, median; PRF, Patient Request Form.

Statistically significant differences (*p* < 0.05) were found between male and female respondents, with women reporting higher scores than men for all three types of expectations measured by the PRF. A low but statistically significant correlation was observed between age and expectations across all scales, with respondents aged 76–90 and 61–75 reporting the highest scores, and those over 90 reporting the lowest. Additionally, a weak correlation was found between education level and expectations, with respondents with secondary or vocational education scoring highest for explanation of illness and test- and treatment-related information, while those with tertiary education scored lowest. Table 3 provides detailed information on the relationships between sociodemographic factors and expectations.

**Table 3:** PRF scores by sociodemographic variables.

Variable		Expectations for explanation of the illness			Expectations for emotional support		Expectations for test- and treatment-related information			
		M	SD	Me	M	SD	Me	M	SD	Me
Sex	Female	6.89	4.57	8.0	4.85	4.24	5.0	7.41	4.73	9.0
	Male	6.47	4.78	7.0	4.35	4.30	4.0	6.81	4.96	8.0
Age	60 or under	6.19	4.76	7.0	3.92	4.01	3.0	6.59	5.0	8.0
	61–75	6.73	4.61	8.0	4.71	4.24	5.0	7.22	4.76	9.0
	76–90	8.34	4.13	10.0	6.51	4.38	6.0	8.79	4.15	11.0
	Over 90	5.79	4.25	6.0	3.88	4.40	1.5	6.75	4.70	7.0
Education	Primary or lower	6.37	4.56	6.0	4.93	4.24	6.0	6.86	4.65	7.0
	Basic vocational	6.95	4.79	8.0	4.90	4.41	5.0	7.34	4.95	10.0
	Secondary/post-secondary	7.06	4.45	8.0	4.84	4.22	5.0	7.64	4.66	10.0
	Tertiary	6.18	4.75	7.0	4.11	4.12	3.0	6.51	4.86	7.0

M, arithmetic mean; SD, standard deviation; Me, median; PRF, Patient Request Form.

### 3.3 Quality of life (SF-36)

The respondents’ mean total SF-36 score was 63.45, indicating a moderate to high quality of life, with moderate variation in scores (SD over 27% of the mean). The mental component summary score (M = 65.07) was slightly higher than the physical component summary score (M = 62.70), with both scores falling in the moderate range. Respondents scored highest in the physical functioning (PF) and social functioning (SF) dimensions, and lowest in vitality (VT) and general health (GH). Most respondents (59.9%) reported high quality of life, while 29.2% reported moderate and 10.9% low quality of life. Table 4 provides detailed SF-36 scores and their distribution.

**Table 4:** Descriptive statistics for the SF-36.

Factor	*N*	M	SD	Min.	Max.	Q25	Me	Q75
Total SF-36 score	2040	63.45	17.681	14.57	95.86	48.29	67.79	78.43
SF-36_physical component summary score	2040	62.70	18.062	14.17	93.96	49.58	66.67	77.92
SF-36_mental component summary score	2040	65.07	19.688	8.18	100.00	44.55	70.45	80.91
PF	2040	71.44	25.245	0.0	100.0	60.0	75.0	90.0
RP	2040	61.75	45.480	0.0	100.0	0.0	100.0	100.0
BP	2040	69.80	18.878	10.0	100.0	55.0	67.5	80.0
GH	2040	42.88	11.584	10.0	85.0	35.0	40.0	50.0
VT	2040	58.02	15.036	0.0	100.0	50.0	55.0	65.0
SF	2040	71.36	22.261	0.0	100.0	50.0	75.0	100.0
RE	2040	67.11	44.955	0.0	100.0	0.0	100.0	100.0
MH	2040	65.81	15.180	20.0	100.0	56.0	64.0	76.0

M, arithmetic mean; SD, standard deviation; Min., minimum value; Max., maximum value; Q_25_, lower quartile; Q_75_, upper quartile; Me, median; SF-36, Short Form Health Survey; PF, physical functioning; RP, role physical; BP, bodily pain; GH, general health; VT, vitality; SF, social functioning; RE, role emotional; MH, mental health.

No statistically significant difference (*p* > 0.05) was found in quality of life scores between male and female respondents, though women had slightly higher total and mental component summary scores, while men had slightly higher physical component summary scores. A moderate negative correlation was found between age and total SF-36 and physical component summary scores, while a weak negative correlation was observed between age and mental component summary scores. Respondents aged 60 or under had the highest total, physical, and mental component summary scores, while those aged over 90 had the lowest. Additionally, a weak correlation was found between education level and quality of life scores, with respondents with tertiary and secondary/post-secondary education reporting the highest SF-36 scores, and those with primary or lower education reporting the lowest.

Table 5 provides detailed information on the correlations between sociodemographic variables and SF-36 scores.

**Table 5:** SF-36 scores by sociodemographic variables.

Variable		Total SF-36 score			SF-36_physical component summary score		SF-36_mental component summary score			
		M	SD	Me	M	SD	Me	M	SD	Me
Sex	Female	63.53	17.18	67.71	62.66	17.67	66.77	65.43	19.21	70.45
	Male	63.26	18.76	69.07	62.81	18.91	66.25	64.26	20.70	70.00
Age	60 or under	71.90	12.86	74.29	71.70	13.33	75.42	72.34	15.12	74.55
	61–75	61.47	17.59	62.43	60.87	17.51	60.00	62.78	20.41	66.82
	76–90	52.17	18.43	49.57	49.86	18.74	48.96	57.20	20.97	55.91
	Over 90	49.05	20.33	52.64	45.14	21.33	44.38	57.58	21.68	62.73
Education	Primary or lower	55.63	17.58	54.43	53.68	18.45	53.13	59.89	19.78	64.55
	Basic vocational	62.21	18.27	64.86	61.65	18.66	63.13	63.44	20.10	68.64
	Secondary/post-secondary	63.10	17.07	66.36	62.35	17.18	65.10	64.73	19.72	70.23
	Tertiary	68.29	16.71	73.43	67.78	17.12	73.75	69.39	18.32	73.64

M, arithmetic mean; SD, standard deviation; Me, median; SF-36, Short Form Health Survey.

### 3.4 Correlations between patients’ expectations of health professionals and their quality of life

There was a statistically significant correlation between expectations for explanation of the illness and several SF-36 dimensions, including physical functioning (PF), bodily pain (BP), social functioning (SF), general health (GH), and the total SF-36 score. Higher expectations for explanation of the illness were associated with higher scores for general health, social functioning, and mental health (MH), but lower scores for physical functioning, role limitations (RP), and bodily pain. A medium correlation was observed between expectations for emotional support and physical functioning and total SF-36 scores, with higher expectations for emotional support associated with lower scores across all SF-36 subscales. Expectations for test- and treatment-related information were also correlated with physical functioning, social functioning, and mental health, with higher expectations linked to better social functioning, mental health, and vitality, but poorer physical functioning and bodily pain. Table 6 provides detailed correlation data.

**Table 6:** Correlations between scores for expectations for explanation of the illness, expectations for emotional support and expectations for test- and treatment-related information and scores on the SF-36.

Factor		PF	RP	BP	GH	VT	SF	RE	MH	SF-36
Expectations for explanation of the illness	r	−0.194	−0.067	−0.112	0.057	0.019	0.128	−0.003	0.062	−0.082
	*p*	0.000^*^	0.002^*^	0.000^*^	0.010^*^	0.401	0.000^*^	0.900	0.005^*^	0.000^*^
Expectations for emotional support	r	−0.317	−0.237	−0.245	0.043	−0.176	−0.178	−0.228	−0.222	−0.317
	*p*	0.000^*^	0.000^*^	0.000^*^	0.055	0.000^*^	0.000^*^	0.000^*^	0.000^*^	0.000^*^
Expectations for test- and treatment-related information	r	−0.167	−0.024	−0.077	0.038	0.060	0.183	0.042	0.105	−0.035
	*p*	0.000^*^	0.285	0.001^*^	0.083	0.007^*^	0.000^*^	0.060	0.000^*^	0.110

SF-36, Short Form Health Survey; PF, physical functioning; RP, role physical; BP, bodily pain; GH, general health; VT, vitality; SF, social functioning; RE, role emotional; MH, mental health; p, p-value; r, Spearman’s rank correlation coefficient.

* Statistically significant.

## 4 Discussion

### 4.1 Expectations of health professionals

The present study showed that what the Płock residents aged 55 and over surveyed expected most from health professionals was test- and treatment-related information (mean score on the PRF: 7.22 ± 4.809), followed by explanation of the causes and consequences of their illness (mean score on the PRF: 6.76 ± 4.638). The least commonly reported expectations were for emotional support (mean score on the PRF: 4.69 ± 4.263). These findings are largely consistent with those from other studies in the literature. In a study by Kurowska et al. (21), which was conducted using the Polish version of the PRF, the patients included in the study reported the highest scores for expectations for explanation of the illness (mean score: 9.02 ± 3.385) and expectations for information about tests and treatment (mean score: 9.0 ± 3.811). The respondents reported the lowest scores for expectations for emotional support (mean score: 4.72 ± 3.957). Similarly, in a study by Cieślak et al. (22), the most commonly reported expectations were for explanation of the illness and information about tests and treatment, and the least commonly reported expectations were for emotional support. Similar results were reported by Cybulski et al. (23) in their study using, among other instruments, the Polish version of the PRF. The older adults included in the study were least likely to seek emotional support (mean score: 5.11 ± 4.01). Expectations for explanation of the illness were more commonly reported (mean score: 7.79 ± 4.02). The most commonly reported expectations were for information about tests and treatment (mean score: 8.07 ± 3.87). It can be concluded that the respondents in our study and those included in the studies referred to above were most likely to seek explanation of their illness and information about tests and treatment and least likely to seek any type of emotional support from health professionals. Our findings are consistent with the broader trend observed in similar studies, which indicated that older patients tend to prioritize clear communication and detailed information about their health condition and treatments (18). These expectations are likely driven by the complexity of the medical conditions experienced by older adults, particularly chronic diseases, which necessitate a deeper understanding of medical information to facilitate informed decision-making. On the other hand, the relatively lower emphasis on emotional support suggests that older adults may seek such support elsewhere or may not expect it as part of their interactions with health professionals. This aligns with research by Cybulski et al., who found that older patients prioritize practical information over emotional reassurance, which may be perceived as secondary to their primary medical concerns (23).

In their study using the Polish version of the PRF, Rotter et al. (24) reported a low overall level of expectation for emotional support among the primary care patients studied (mean score: 3.69 ± 0.38). The score for expectations for emotional support reported by the authors was lower compared to that reported in the present study. However, different results have also been reported in the literature. In one study among Lithuanian primary care patients, the ‘emotional support’ factor was found to be one of the four main factors of patients’ expectations (25), whereas in a study by Kurpas et al., the percentage of individuals who felt that their expectations for emotional support from a general practitioner were met was similar to the percentage of individuals who felt they did not receive such support (26).

A study by Kurowska et al. (21) showed that female respondents, respondents aged 41–50, respondents aged over 60 and those with primary or vocational education were more likely to seek explanation of their health problem, emotional support and information about tests and treatment. Similarly, in a study by Cybulski et al. (23), female respondents and respondents aged between 60 and 69 reported higher scores on the PRF.

The present study showed statistically significant differences between men and women in scores for expectations for emotional support and expectations for information about tests and treatment. Different results were reported by Rotter et al. (24), who did not find a statistically significant difference between female and male patients in the extent to which they sought emotional support from their primary care physicians. However, the authors found that the older the patients included in the study were, the more likely they were to seek emotional support and that there was a statistically significant difference (*p* < 0.05) between young individuals and patients in two older age groups in the extent to which they expected emotional support from their primary care physician. A similar finding was made in the present study. Scores for expectations for emotional support increased with age among respondents aged 90 or under. However, respondents aged over 90 had the lowest scores on this scale of the PRF. This may be due to the fact that the proportion of respondents aged over 90 in our study was relatively small and was smaller than proportions of respondents in other age categories. The study by Rotter et al. (24) also found a statistically significant relationship between education and expectations for emotional support. Patients with tertiary education reported the lowest scores on the ‘expectations for emotional support’ scale of the PRF, whereas patients with vocational or primary education reported the highest scores on the scale (*p* < 0.05). The same correlation was observed in the present study. In addition, we found that the lower the education level of respondents, the more likely they were to seek emotional support from health professionals, which is consistent with findings from the study by Rotter et al. (24). There may be several reasons for the significant relationships between sociodemographic factors, particularly age and education, and patients’ expectations of health professionals. The main reason may be that older adults tend to have multimorbidity—they have more health problems than younger individuals and are more likely to be affected by pain. Moreover, they have difficulties in accessing health care. As a result, they have increasing expectations in all the three areas analysed. Older individuals who are lonely and have no peer-aged friends or loved ones who could provide them with emotional support seek such support from health professionals, particularly general practitioners, whom they often trust and even consider friends. Further research is needed to explore whether these expectations are shaped by personal experiences with healthcare or broader societal trends. For example, older adults with lower education levels may feel less empowered to seek detailed medical explanations, relying more on healthcare professionals for emotional support. Conversely, individuals with higher education levels may have greater health literacy, enabling them to independently understand medical information, which reduces their reliance on emotional support from healthcare providers.

### 4.2 Quality of life

Our analysis of the results of the SF-36 showed that the older age Płock residents included in the present study reported the highest scores for the physical functioning (PF) and social functioning (SF) dimensions, and the lowest scores for vitality (VT) and general health (GH). In a study by Lövkvist et al. (27) among Swedish women with endometriosis, the participants reported significantly lower scores on the subscales of the SF-36, particularly on the vitality (VT), role physical (RP) and general health (GH) subscales. In a study by Payne et al. (25), respondents reported the highest scores for social functioning (SF) and mental health (MH), and the lowest scores for vitality (VT) and physical functioning (PF). The Tehran residents aged 55 and over included in a study by Tajvar et al. (28) reported the highest scores for bodily pain (BP) and social functioning (SF), and the lowest scores for role limitations due to physical health problems (RP) and role limitations due to emotional problems (RE). The scores reported in that study were significantly lower compared to those reported in the studies referred to above. In a study by López-Ortega et al. (29), the older individuals included in the study reported the lowest scores for vitality (VT), general health (GH) and mental health (MH), and the highest scores for role limitations due to emotional problems (RE), role limitations due to physical health problems (RP) and social functioning (SF). Similar findings were reported in studies by Polish authors. In a study by Knapik et al. (30), the older adults surveyed reported the highest scores for social functioning (SF) and physical functioning (PF), and the lowest scores for role limitations due to physical health problems (RP) and general health (GH). In a study by Mirczak (31) among older people living in rural areas, study participants reported the highest scores for the role emotional (RE) and mental health (MH) dimensions of the SF-36, and the lowest scores for the physical functioning (PF) and role physical (RP) dimensions of the questionnaire. Findings from the present study show that the older adults surveyed reported significantly higher scores for the physical domain than for the mental domain of HRQoL. One exception were respondents living in rural areas, who reported higher scores for the mental domain than for the physical domain.

Our study showed no significant difference in quality of life between male and female respondents (the scores reported by men and women were almost identical). Men reported slightly higher physical component summary scores. In a study by Mirczak et al. (31), the authors found significant differences between male and female respondents in the physical functioning domain of HRQoL, with men reporting higher scores for this domain. Most studies in the literature on the subject show that women tend to report poorer HRQoL than men (32–36). In a study by López-Ortega et al. (32), female participants reported lower scores in all dimensions of the SF-36, apart from general health, with the largest difference seen in the physical functioning dimension. Differences between men and women in scores for physical functioning, vitality and bodily pain were statistically significant (*p* ≤ 0.01). The fact that women report poorer HRQoL may be due to their anatomical, physiological and psychosocial characteristics (37, 38). In their study, Guallar-Castillón et al. (33) noted that the fact that the women included in their study reported lower quality of life scores than men may be due to the female participants having a lower educational level and a higher BMI and engaging in less physical activity compared to male participants.

In line with previous research, our study found a relationship between the quality of life of older adults and their age and education (39–41). Similarly, López-Ortega et al. observed a decline in quality of life with age, with significant differences between respondents aged 80 and above and younger cohorts, particularly in the physical functioning and role physical dimensions (29). Moreover, studies such as Aghamolaei et al. (36) support our findings that higher education is associated with better overall quality of life, emphasizing the role of education in shaping health outcomes and perceptions among older adults. We found that quality of life as measured by the SF-36 decreased with age and increased with increasing education and that there was a statistically significant low correlation between the level of education of the respondents and their total SF-36 scores (*p* < 0.001), physical component summary scores (*p* < 0.001) and mental component summary scores (*p* < 0.001). Similarly, in a study by López-Ortega et al. (29), scores in all dimensions of the SF-36 decreased with age, with the largest differences seen between respondents aged 80 and over and other age groups. The study found statically significant differences between age groups in scores for the physical functioning (*p* < 0.05), role physical (*p* < 0.001), bodily pain (*p* < 0.001) and general health (*p* < 0.05) dimensions.

Our results are consistent with the findings of Kurowska et al., which showed that older patients, particularly women, tend to have higher expectations for clear communication and medical information (21). However, Rotter et al. (24) did not observe significant gender differences in emotional support expectations, suggesting that patient demographics and cultural factors might influence these expectations differently across populations.

### 4.3 Limitations

Our study has certain limitations. Firstly, our findings are based on the assessment of the subjective feelings of the older adults surveyed. The study used standardised scales, which are sensitive research tools. However, they are based on the self-report of subjective feelings, as opposed to objective criteria, which can result in false positive findings. Secondly, even though our sample was large, it included residents of only one city. Therefore, it is difficult to apply the results of the study to the general population of older people in Poland, and it is even more difficult to apply them to the population of older people living in rural areas. Thirdly, women were overrepresented in our study. Therefore, our findings should be tested in a study including an equally large group of men. However, the majority of the population of older people in Poland are women (older women are overrepresented compared to older men), which is a significant problem. Despite these limitations, our findings may serve as a starting point for further research on satisfaction with life and preferred behaviours among older people in Poland and their sociodemographic characteristics. Ideally, the research should have the form of a longitudinal nationwide study.

## 5 Conclusion

A number of important conclusions can be drawn from the present study on Polish older adults’ expectations of health professionals and their quality of life. The study showed that what the older adults surveyed expect most from health professionals is accurate medical information, particularly information about their health. Respondents in our study reported moderate, bordering on high, overall quality of life. Specifically, females and younger respondents reported significantly higher expectations for clear communication and emotional support compared to males and older respondents. Likewise, individuals with tertiary education demonstrated a notably higher quality of life, particularly in the mental and physical health domains, compared to those with lower educational levels. These findings suggest that sociodemographic characteristics play a key role in shaping expectations towards healthcare professionals and perceptions of quality of life. We found that the quality of life of the older adults surveyed and their expectations of health professionals were influenced by sociodemographic variables. Female respondents, younger respondents and respondents with tertiary education reported a higher level of expectations and higher quality of life. One exception were respondents aged 76–90, who reported the highest level of expectations. There was a statistically significant correlation between the respondents’ scores for expectations for explanation of the illness and expectations for emotional support and their overall perceived quality of life. Our findings highlight the need for further more in-depth research on a larger scale in Poland to gain a better understanding of the phenomena and their possible correlations.

## 6 Practical implication and future direction

These results emphasize the importance of adapting healthcare communication strategies to meet the diverse needs of older patients. Women and individuals with lower educational levels may benefit from enhanced emotional support during interactions with healthcare professionals, whereas those with tertiary education may require more comprehensive and detailed medical information. By recognizing and addressing these varying expectations, healthcare providers can improve patient satisfaction, increase adherence to medical recommendations, and ultimately enhance the overall quality of care for older adults. Future research should investigate how these findings generalize to different regions of Poland, particularly rural areas, where access to healthcare and patient expectations might differ. Additionally, longitudinal studies are needed to explore how patient expectations and quality of life evolve over time, especially as health conditions change and as older adults continue to interact with healthcare systems.

## Data Availability

The raw data supporting the conclusions of this article will be made available by the authors, without undue reservation.
